# Chronicles of the Octopus*This appeared in the January number and created a sensation. The demand for it exceeded the supply and soon exhausted the edition; hence I reproduce it here. I regret that I cannot reproduce the pretty little half-tone cut of Dr. Geo. H. Simmons’ newspaper advertisement of his “Lincoln (Neb.) Medical Institute and Water Cure Establishment,” which accompanied this article in the January number. I lent it to a fellow for his February number

**Published:** 1909-02

**Authors:** 


### CHRONICLES OF THE OCTOPUS.*

### EVOLUTION OF THE GREAT MEDICAL TRUST.

"Big aches from little toe-corns grow."—*Shakespeare.*

And it came to pass in those latter days that in the city called She Cargo (which being translated means "The Windy"), there dwelt a mighty man of ethics, whose name was Simon, surnamed The Boss. He was a wayfaring man from beyond the seas, aye, even from the country called Ireland (which being translated means the land of wrath), in the region round about Dublin, and was of the tribe of Hahneman-the-Homo. He blew into The Windy from the wilderness of Nebraska, even from the city of Lincoln, where he preached the gospel of Homeopathy and Water Cure for all the ills that flesh is heir to, from sore eyes to salpingitis, and proclaimed it from the housetops through a mighty trumpeter called *The Nebraska State Journal,* a secular sheet, in its Sunday editions.

Now it came to pass that in the year eighteen hundred and eighty-eight, or thereabout, this Apostle having waxed exceeding weak from such slim diet and the inability to gather much shekels, shook the dust of the wilderness off his feet, and, girding up his loins,—without purse or script,—journeyed unto the City of the Wind (and likewise of graft, trusts and combines), and there raised his Ebenezer.

Now this Simon took counsel with himself how best he could "raise the wind" and, incidentally, raise hell generally. He said: "Behold, am I not a versatile man, and can not I, like Paganini, play all tunes on one string? Can I not be anything that pays? Whether it be Homo, Osteo, Physio-Med or Regular? By the beard of Hahneman, the prophet, I'll do it. I'll be a Reformer for sure. For—behold, are the pathies not all our brothers; aye, verily, the Regulars (of the Tribe of Hippocrates), the Galenites and the Thompsonians (the disciples of The Herbes), the Osteos of the Tribe of Still-ites; aye, even the Ethiops of the Tribe of

*This appeared in the January number and created a sensation. The demand for it exceeded the supply and soon exhausted the edition; hence I reproduce it here. I regret that I cannot reproduce the pretty little half-tone cut of Dr. Geo. H. Simmons' newspaper advertisement of his "Lincoln (Neb.) Medical Institute and Water Cure Establishment," which accompained this article in the January number. I lent it to a fellow for his February number

Sambo? These be all brothers, and I will gather them into the fold of the Octopus, and I shall reap much shekels. Moreover, are they all not divided on Therapeutics (which being translated means how best to bamboozle the public and gather coin)? And are not they—many of them, aye, even the best of them, gone astray, running after strange drugs called proprietaries, and tooting wind instruments—privately-owned-and-run-for-profit? Are they not hankering after the fleshpots of the Antis, and the 'Ines,' polluting the air and defiling the temple of the A. M. A. with the breath of the Whore of Babylon, called Old Auntie Komenigh-us? Aye, verily. That same shall they not do." And he vowed a vow.

Straightway this great Simon summoned his principal adviser, one Priestly—a countryman of his—and took counsel of him, saying: "How shall I get control of that Monster Machine called The Octopus?" "Dead easy," says the High Priest(ly). "I'll put up a job on the Trustees. They shall fix the salary at such a figure that no American physician can accept the position of Boss (which is to give notice that no American need apply), and trust to luck to get a raise, later." And so it came to pass.

Now, being full-fed and flush, Simon waxed exceeding strong. He straightway called together his Council of Pharmacy (most of whom never saw a "pharm"), and he said: "Thus say I, The Boss: It shall not be lawful, or ethical, to advertise, dispense, sell, give away or prescribe any drug or combination not approved by my Board. See to it. Give notice to the Scribes and Pharisees of the tribe of Editors and the sect of Independents—so-called—that they shall not advertise any of the 'antis' or 'ines,' or trade-marked preps (unless the name be changed to conform to the U. S. P. and N. F. and N. A. R. P., for their substitutes, under Latin names are 'just as Gude,' but do not smell so good), and we will thus cut off the revenues of the run-for-profit heretics, who dare lift up their piping voices against *me*—even me, The Boss. And they shall perish from the earth forever. Selah!"

And lo, that all might hear the glad tidings of a newer ethics from the oracle and lawgiver of the Octopus, he summoned his Chief Spieler, one Mike Cor, of the Tribe of the Micks, and said: "Go thou into all the land, even unto Texas and the country round-about Austin, and preach the gospel of Unification to all 'Docs' of whatever persuasion,—the Allos, the Homos, the Physio-Meds, the Eclectics, the Osteos (called also the Still-ites), aye, even to the

Ethiops of the Tribe of Sambo, for, behold! are they not our brothers?"

And the Chief Spieler, sometimes called The Walking Delegate, departed and journeyed into all the States, even unto Texas. He lifted up his voice in the Synagogues and in the county societies, and in the market places, crying aloud, "Organize! Organize! Round up the Docs! Corral them. Let none escape; no, not one, for he who sent met is greater than I, and he would have a great Trust, and get a corner on subscriptions for The Octopus. Every son of Aesculapius,—every son of Hahneman and of Still and of Galen and of Thompson,—every son of Ham of the Tribe of Sambo,—aye, every son-of-a-gun corraled, means five shekels a year, and they will get a copy of the great Octopus Organ weekly, whether they want it or not. Let none escape, under penalty of denunciation of being a scab and not in good standing." Thus saith Simon, The Boss.

And straightway Mac-the-Mick appointed captains of tens and captains of hundreds, called Councilors, and behold! the sects of Independents and their chief scribes—not seeing the intent—joined in with one voice and cried "Organize." And it came to pass that in each State there was a shaking up of dry bones, and every fellow hastened to enroll; for the fear of being a scab was heavy on them. Aye, the Independent-personally-owned-and-run-for-profit layouts all joined in the chase, and, like faithful collies, rounded up the "Docs" and drove them, like sheep to the shambles to be shorn; aye, like faithful deer hounds, they tracked the quarry for The Boss, who unheeding of the fate of Actæon did not foresee the day, now come, when those *hunting dogs* should turn upon and rend him.

And it came to pass that in addition to the captains of tens and captains of hundreds, called Councilors, there was appointed in each State a Captain of Hosts, who should be Chief Scribe and Keeper of the Records and Seal, and gather in the tribute. And every "Doc" in the land was made to stand and deliver, once a year, a tax of two shekels, and receive monthly a State Tooter, sometimes called a Tentacle, whether he wanted it or not. Now these Tooters were to echo the toots from the Tooter-in-Chief of ye Octopus, under penalty of going scalpless to bed. And the alacrity with which some of them obeyed was diverting. The old friends, the scribes of former years who had been faithful, efficient and loyal, were to be kicked out, and it was advertised that they were "unclean and infectious and unworthy of support."

And the Chief Spieler departed and again journeyed even unto Texas. He lifted up his voice in the Synagogues and the county societies and in the market places as before, and cried with a loud voice: "Repent ye, for the day of unification of all 'schools' and of mixed Boards is at hand." And certain ultra ethical disciples of Hippocrates and of Aesculapius,—aye, even of the Divine Apollo, who, in that ancient day (a few years ago) despised and reviled these sectarians, calling them quacks, and poked out their tongues at them in derision—fell over each other in their eagerness to embrace the Spieler.

And it came to pass that there was gathered together in the city called Atlantic, hard by the sea and nigh unto the shores of Jersey, a great multitude whose numbers were as the sands of the shore. Black, white and tan,—Allo, Homoeo, Osteo, Samb(e)o; and at the sound of the trumpet in the hands of the Chief Spieler—Mac-the-Mick, they all fell down and worshiped, saying, "Ain't he just the Real Thing?" And straightway The Boss caused a proclamation to be proclaimed, saying, "Be it known to all men that The Great Octopus, whose prophet I am (and there is none other), orders that no individually-owned-and-run-for-profit concern carrying unclean and infectious advertisements of the abomination class, called 'Antis,' shall be admitted to The Presence; for are they not unclean? Yea, every one. And are we not holier than they? We, and our Pharmacy Councilors and our Chief Tooters and Toadies? Selah. I have said."

Straightway all the scribes of the editorial sects of the Independents, when they heard these things, assembled together and took counsel, the one with the other. Much they marveled and were sore afraid. They lifted up their voices and wailed a big wail, that was heard from Dan to Beersheeba,—the distance of a day's journey. "What manner of man is this?" said he of the *Cricket and Guy*. "On what meat doth he feed that he is grown so great? Is it not he who preached water cure, with homeopathy on the side, in the wilderness of Nebraska? Yea, verily! And did he not advertise in certain abominations called lay papers? Yea, in the Sunday editions thereof, after the manner of the quack and the charlatan? And is it he who now would instruct us, even us, the elect, in ethics? Go to. It shall not be. Have we not pulled the paps of dear Old Antie Kam, lo, these many years, and waxed fat? Did she not give us suck when we were puling babes? And shall we now turn upon her and cast her into outer darkness?" And there was weeping and wailing and smashing of teeth.

But there were those amongst them who waxed bold and were exceeding wroth. These went unto their several sanctums scattered in the cities from the North to the South, from the East to the West,—even from Portsmouth to Charleston by the sea, and Savannah, and from New York to Texas, from the cities of the Middle West to the Gulf; and these doughty Knights of the Independent-run-for-profit-personally-owned concerns rashly fired dynamite cartridges at him—at long range—on the murderous toy pistol, so to speak, and even dared to shoot editorial squibs at him, and throw deadly spit balls at the Tyrant! They heaped anathemas on him. They reviled him and called him names. They wagged their heads and uttered cuss words. But, they all anted their yearly tithe of five shekels and *staid in,*—not even calling for a new deal.

Now it fell out that in the City of the Wind,—even of the She Cargo,—there dwelt certain Philistines of the tribe of The Live Wire and of the Square Deal. They took counsel of each other, chief amongst whom were G. Whiz Lydston, the Hard Hitter, and Abbott, surnamed the Strenuous, Chief of the Editorial Scribes and of the Square Dealers, and Robinovitch of the *Cricket and Guy.* These rose up and smote the Tyrant, hip and thigh, so that he was sore rattled; and he cried aloud to his man Friday (whose name is Jonesey, surnamed the Joke), Captain of the Hosts on the Golden Horn—even at the City of San, saying, "The Philistines be upon me, Jonesey, my Boy; whither shall I flee? Verily, they are after my scalp, and I am sore afraid."

And Jonesey answered him, saying, "Be not afraid, for lo, I am with you always. Know ye not that I am the same kind of a hairpin that you are? Have I not caught on, like you, after trying all things and holding fast to none? Have I not been, first and last, a jack of all trades, succeeding at none; a specialist for everything under the sun, but never till the day you discovered me have I struck pay gravel. I'm with you. Brace up."

And Simon was comforted; and they fell each upon the other's neck and wept tears. Yea, verily, they wept a great weep.

And it came to pass that when the King (no, the Boss, I mean) had assembled his wise men and his Council of Pharmacy and his Tooters of the several Tooting Organs that echoed the big toot, and his Rounder-up, Mac-the-Mick, and his host of "me-too" claquers, at a great feast in the City of The Wind, lo, there appeared a *handwriting on the wall,* and they were sore afraid and were seized with a great trembling.

And it came to pass that there were none there who could read it, for it was in an unknown tongue.

Now, be it remembered that at that time there dwelt in a far distant country—even in the land of the Long Horns and of the Secesh people, one Daniel, a prophet of great renown in interpreting dreams,—Daniel,—even he of the "Red Back." Him they sent for. And Daniel put on his specs, and, lifting up his voice, cried with a loud cry:

"MENE, MENE (Min-e-mo)
TEKEL (Me and I'll) TEKEL (You).
UPHARSIN!"

"Behold! *A House Divided Against Itself Must Fall. Thou Art Weighed in the Balance and Found Wanting.* Get thee back to the wilderness of Nebraska; yea, even unto thy Water Cure Institute; for we want thee not." Thus saith Daniel, the prophet. Selah!

---

### ESPECIAL NOTICE TO MY EXCHANGES.

Look at your list, and if you are sending your journal (in exchange for the "Red Back") to Dr. Russ at San Antonio, *stop it,* and send it to me at Austin. I requested this several years ago, but some of my most valued exchanges failed to make the change, and I "have not seen the hair nor the hide of 'em in years." Do it now.

---

### A STATE BOARD OF HEALTH FOR TEXAS.

Below I append an official synopsis of the bill to abolish the present antiquated "health department" and create a State Board of Health. (The "Red Back" is the first to publish this, as usual.) The bill has been carefully prepared by the Legislative Committee of the State Medical Association in collaboration with the State Health Officer, Dr. D. R. Fly, Dr. M. M. Carrick, and Special Attorney Wilkinson. It was introduced in the Senate on the 12th of January by Senator Harper and in the House by Colonel Ralston. That it shall have the cordial, earnest and unqualified support of the "Red Back" "goes without saying."

"This is the way I long have sought and mourned because I found it not."

The bill proposes to abolish the present State Health and Vital Statistics Department and to create instead a Board of Health.

It is to be composed of seven members, five of whom shall be physicians legally qualified to practice medicine within the State, graduates of reputable medical schools. One member to be designated by the Governor "Pure Food Commissioner," who shall be a chemist and bacteriologist. One member to be Live Stock Sanitary Commissioner.

The President of the Board is to be designated by the Governor State Health Officer, to be appointed by the Governor and confirmed by the Senate; to receive a salary of $3600 per annum. The Pure Food Commissioner and Live Stock Sanitary Commissioner to receive the sum of $2500 each per annum.

The Board to be a body corporate, having a seal, and with power to sue and be sued.

The Board shall employ an Assistant Health Officer and three inspectors. The Assistant Health Officer to receiver $2000 per annum; the inspectors $1800; also one bacteriologist and chemist at $1800, who shall be under the direction of the Board and assist the Pure Food Commissioner.

Four members of the Board to receive no salary but to have an allowance of $10 per day while in session for expenses. Shall also have a registrar of vital statistics at $1800 per annum. A majority of the Board shall constitute a quorum for the transaction of business. The Board to meet on call of the President or the demand of three members in writing.

The Board shall have offices in the capitol.

The President of the Board to execute bond in the sum of $10,000.

The Board to have general supervision and management of the Live Stock Sanitary Commission, but that department is to be run under existing laws, and have separate divisions of work. The same as to the Pure Food Commission.

The President of the Board to be chief quarantine officer of the State, but the quarantine service is to remain on its present operating basis.

The Board shall be authorized to enact an up-to-date sanitary code for Texas, to embrace all matters of health and disease prevention, sanitation and school hygiene; to regulate the manner and method of the interment, disinterment and transportation of dead bodies; to regulate all matters of internal quarantine. And to provide for the health and safety of the people of Texas in all matters of inspection and general sanitary conditions.

The code when adopted by a majority of the votes of the Board is to be published for three weeks in three daily papers of the State, and shall then become law. To carry penalties not in excess of $500 for violation. The Board is to print and publish a sufficient number of copies for general distribution; to be furnished free to all State and county and city officials. To be paid for at a nominal sum to cover cost of publication and carriage by the general public.

All the members, officers and inspectors of the Board are to be constituted peace officers, and to have the power of arrest for violation of ordinances of the code or for violation of quarantine laws, when executing warrants of arrest duly issued by a proper officer.

The Board to have power to summons witnesses to investigate and abate nuisances.

The bill creates county and city health officers in all the organized counties, and in all the incorporated cities and towns within the State, and makes it the duty of such officers to co-operate with the Board of Health in all matters of public health promotion.

The bill provides for an annual conference of county and city health officers.

---

### The January "Red Back" was a Whizzer. They say: Vesuvius in Eruption.

DALLAS, TEXAS, January 16, 1909.

*Dr. Ferdinand Eugene Daniel, Editor of the "Red Back," Austin, Texas.*

DEAR DOCTOR: Since the earthquake, shaking the foundations of Italy and Sicily; with the periodic tremors of this sphere felt in that locality since, and with the disturbance observed at Mt. Vesuvius, we have been daily expecting another eruption; one possibly that will surpass anything known in the history of this monster volcano. Now, from the latest "Red Back" I see that old Mt. Vesuvius is now pouring forth smoke and lava, throwing high into the air immense boulders, which are rolling down its sides and will undoubtedly cause the people living in the city of "She-Cargo," as well as "Simon" and his brother, "Mac-the-Mick," to hie themselves to the "Golden Horn" and seek shelter with one

"Jonesy." I have read your editorial, and I just want to say that *it was great.* Where did you get the advertisement of the Lincoln Medical Institute?

As soon as I finish my morning's work I shall take up in detail Dr. G. Whiz Lydston's article.

How are you? I wish you a Happy and Prosperous New Year, and I shall certainly see you and Mrs. D. at the State Medical Association in Galveston.

Remember, your speech defeated Dallas for the meeting last year, but I now write to ask for a "five-minute's waggle of your silver tongue" in our behalf at the Galveston meeting. Do not forget it. Dallas wants and is entitled to the 1910 meeting. Selah!

I remain,

Yours fraternally,

M. M. SMITH.

---

ST. LOUIS, MO., January 16, 1909.

*Editor Texas Medical Journal.*

DEAR DOCTOR: Enclosed find check for $5.00 (for the next five years' subscription to the "Red Back"). Go after 'em; wake 'em up; make Rome howl, and make the fur fly. All the world loves a lover (and a fighter). Daniel, Lydston and Abbott—Gee! there'll be some "diddings," I'm thinking, when you talk out loud. Burn 'em alive; sizzle and fricassee 'em, and damned be he who first cries "hold, enough!"

Yours truly,

OLIVER O'BAR.

---

MONTAGUE COUNTY MEDICAL SOCIETY.

OFFICE OF L. P. TENNEY, M. D., SECRETARY-TREASURER.

STONEBURG, TEXAS, January 20, 1909.

*Editor Texas Medical Journal, Austin, Texas.*

DEAR DOCTOR: The "Red Back" has been a welcome visitor to my office for the past ten years, and it is with much pleasure that I look forward to its arrival each month. Of late, however, my interest in its coming has been doubly keen, and let me say the January number is *undoubtedly the best thing I have ever had the pleasure of reading.* The editorial, "Evolution of the Great Medical Trust," is worth the price of the JOURNAL for five years. Dr. Lydston's "Medical Politics" is also a "peach."

How long! how long, will the medical profession lie asleep and allow themselves to be driven into any old thing that suits the management of the Octopus? Where is our independence? If your editorial in the January number doesn't cause something to be a-doing, I am at a loss to know what will.

Keep up the good work; never cease hammering the Octopus, and the profession of Texas will some day "rise up and call you blessed."

Fraternally,

L. P. Tenney.

---

Recognition of the Irregulars.—I told you so. When, influenced by the propaganda promulgated by the apostate homeopath misfit, in control of the Octopus, and preached by the Chief Spieler, "Mac-the-Mick," our State Medical Association, through a clique in control of the House of Delegates, consented to affiliate on a Board of Examiners with homeos,—osteos, physios and eclectics, and two of our ex-Presidents accepted service on that board, the "Red Back" warned the profession that, having let down the bars, there would be no stopping the entrance of all kinds of cattle, and no place to draw the line. It has come to pass. These mixed fellows are now demanding "recognition" on the State Board of Health now being sought at the hands of the Legislature now in session, and they openly threaten to "kill the bill" if not given a place on it. And the homeos have a bill before the Legislature (introduced by Senator Cofer, of Cooke county) providing for a chair of homeopathy in the Medical Department of the University of Texas. If this is conceded, next Legislature will be asked to provide chairs of osteopathy, physioapathy, eclectio-apathy and Sambo-apathy.

How are the mighty fallen! Less than ten years ago the Texas State Medical Association unanimouly adopted a resolution (by Britain) to "expel any member who would so lower the dignity of the profession as to consult with a homeopath"; and in 1884, at Milwaukee, the A. M. A. refused to seat delegates from New York who favored affiliation with homeopaths. And in 1877 (I have the record) the State Association of Texas moved heaven and earth, so to speak, to expel old Dr. Ross, of Brenham, for serving on a local board with a homeopath. We will see that the next demand will be for representation on the Board of Regents of the University; for representation on the medical staffs of our eleemosynary institutions, and, then, the State Health Officer will have to be a homeo or an osteo, or some other kind of off-color fellow.

It was a grave error of judgment—most deplorable—that our Association permitted itself to be wheedled—or bulldozed—into consenting to a *Mixed Board.* It is a failure. It lets the quack out every time. As a matter of fact, the Association *did not* consent to it. Under our organization, 95 per cent of the membership have no voice in anything; they are disfranchised. The House of Delegates run the machine, and many delegates dare not oppose the clique in control, who are under the domination of the Octopus.

---

Dr. A. W. Fly, of Galveston, has been appointed a member of the Board of Regents of the University. The medical profession and the people of Texas, have cause for congratulation on this most excellent selection by the Governor; for Dr. Fly is a most "exceptional man" in point of intelligence, learning, skill in his profession, personal popularity, and general fitness for this public service. He is a strong personality. He is a man of excellent judgment, and is fearless in his convictions. We have now two representatives of the medical profession on this board; two of our most representative and typical men—McLaughlin and Fly.

